# Metabolomic insights into system-wide coordination of vertebrate metamorphosis

**DOI:** 10.1186/1471-213X-14-5

**Published:** 2014-02-05

**Authors:** Taka-Aki Ichu, Jun Han, Christoph H Borchers, Mary Lesperance, Caren C Helbing

**Affiliations:** 1 Department of Biochemistry and Microbiology, University of Victoria, Victoria, BC V8W 2Y2, Canada; 2 University of Victoria – Genome British Columbia Proteomics Centre, Vancouver Island Technology Park, Victoria V8Z 7X8, BC, Canada; 3 Department of Mathematics and Statistics, University of Victoria, Victoria, BC V8W 3R4, Canada

**Keywords:** Postembryonic development, Thyroid hormone, Metamorphosis, Metabolites, Serum, Ultra-performance liquid chromatography, Quadrupole time-of-flight, Mass spectrometry, Vertebrate

## Abstract

**Background:**

After completion of embryogenesis, many organisms experience an additional obligatory developmental transition to attain a substantially different juvenile or adult form. During anuran metamorphosis, the aquatic tadpole undergoes drastic morphological changes and remodelling of tissues and organs to become a froglet. Thyroid hormones are required to initiate the process, but the mechanism whereby the many requisite changes are coordinated between organs and tissues is poorly understood. Metabolites are often highly conserved biomolecules between species and are the closest reflection of phenotype. Due to the extensive distribution of blood throughout the organism, examination of the metabolites contained therein provides a system-wide overview of the coordinated changes experienced during metamorphosis. We performed an untargeted metabolomic analysis on serum samples from naturally-metamorphosing *Rana catesbeiana* from tadpoles to froglets using ultraperformance liquid chromatography coupled to a mass spectrometer. Total and aqueous metabolite extracts were obtained from each serum sample to select for nonpolar and polar metabolites, respectively, and selected metabolites were validated by running authentic compounds.

**Results:**

The majority of the detected metabolites (74%) showed statistically significant abundance changes (*p*_adj_ < 0.001) between metamorphic stages. We observed extensive remodelling of five core metabolic pathways: arginine and purine/pyrimidine, cysteine/methionine, sphingolipid, and eicosanoid metabolism and the urea cycle, and found evidence for a major role for lipids during this postembryonic process. Metabolites traditionally linked to human disease states were found to have biological linkages to the system-wide changes occuring during the events leading up to overt morphological change.

**Conclusions:**

To our knowledge, this is the first wide-scale metabolomic study of vertebrate metamorphosis identifying fundamental pathways involved in the coordination of this important developmental process and paves the way for metabolomic studies on other metamorphic systems including fish and insects.

## Background

After embryogenesis, many organisms experience obligatory developmental transitions to successfully move from one ecological niche to another. One such transition is through metamorphosis in which an immature larva transforms into a juvenile or adult scarcely resembling the initial form. Classic examples occur in vertebrates and invertebrates alike, and often require the involvement of hormone signaling systems. However, a fundamental question in biology remains in understanding how a fully-differentiated organism coordinates the many tissue- and organ-system changes during the metamorphic process [1,2].

Frog tadpoles undergo significant morphological changes, resulting in the development of limbs, resorption of the tail, and a shift from gill to lungs in respiratory organs used, hence a shift from purely aquatic to a semi-terrestrial lifestyle and a change in diet. This extensive process involves apoptosis, cell proliferation, and reprogramming and highlights the complexity, tight regulation, and interconnection of biological networks and pathways.

Despite such complexity, anuran metamorphosis is initiated solely by thyroid hormones (THs) and this important postembryonic developmental period can be divided into three specific stages: premetamorphosis, prometamorphosis and metamorphic climax, characterized in part by TH status [2]. Premetamorphosis is the period after embryogenesis in which free-living tadpoles increase in size in the absence of THs. During prometamorphosis, endogenous TH levels start to increase, causing morphological changes such as the growth of the hind limbs. Metamorphic climax is characterized by the highest level of THs and drastic morphological changes including the complete resorption of the tail and the formation of a stomach.

Metabolomics is the comprehensive analysis of the whole metabolome (metabolite profiles) under a given set of conditions [3] and is a burgeoning field that has started to play a crucial role in systems biology and personalized medicine [4,5]. Metabolomics differs from other "omics" tools in many ways. The metabolome directly represents the phenotype unlike the genome, transcriptome or proteome, the dynamic range is much wider, and the metabolome is far more chemically heterogeneous and complex, thereby producing large, complex datasets that require rigorous computational and statistical analyses [6,7]. Despite these challenges, the direct link of the metabolome to the phenotype is an advantage because genomic or transcriptomic changes may or may not affect the protein level, and proteomic changes may or may not affect metabolites [8].

To our knowledge, no comprehensive metabolomic study has yet been conducted on metamorphosis [9]. We applied a global, mass spectrometry (MS)-based metabolomics approach, using ultra-performance liquid chromatography (UPLC) coupled to a quadrupole time-of-flight (Q-TOF) mass spectrometer, to identify metabolites in serum samples from *Rana catesbeiana* (North American bullfrogs) at different postembryonic developmental stages: from tadpoles to froglets. Serum was the tissue of choice to provide an overall view of the dynamic changes experienced by the frog tadpole and enable the identification of metabolites involved in the coordination of metamorphic processes throughout the tadpole. *R. catesbeiana* were used in the present study because of their large size enabling the analysis of serum from individual animals, their world-wide distribution and availability, and their genetic diversity and life history resemble that of humans more closely than other laboratory frog species [9,10]. In fact, anuran metamorphosis is developmentally equivalent to postembryonic organogenesis in mammals [11]. Both systems share considerable similarities in general processes (cell proliferation, differentiation, and apoptosis), biochemical and molecular events (a switch from fetal/larval to adult hemoglobin in red blood cells, skin keratinization, and urea cycle enzyme induction) and, most strikingly, the developmental progression of structures and functions in the central and peripheral nervous system [2,11].

We show herein that substantial fluctuations in metabolite abundance and extensive remodelling in metabolic pathways occur during *R. catesbeiana* metamorphosis. In particular, we observed metabolites with a significant abundance change in urea cycle, arginine and nucleotide, cysteine/methionine and lipid metabolism pathways suggesting prominent roles of these pathways in the coordination of the metamorphic process.

## Results and discussion

To discover metabolites with differential abundance patterns and to investigate the developmental changes in the metabolic pathways of *R. catesbeiana* during metamorphosis, *R. catesbeiana* tadpoles were divided into seven different developmental stage ranges based on Taylor and Kollros [12] (TK) stages: VI–X, XII–XV, XVI–XVII, XVIII, XIX–XX, XXI–XXII, and > XXV. Twelve samples, each from an individual animal, were prepared for each range, yielding 84 samples in total. Serum samples from these tadpoles were obtained by dissection, and to gain a comprehensive overview of the profile of metabolites, two types of extracts were prepared for the subsequent MS analyses: "total" (ca. 90% acetonitrile) extracts favoring nonpolar metabolites (using reversed-phase chromatography) and aqueous extracts for polar metabolites (using hydrophilic interaction liquid chromatography). Total extracts were prepared by complete deproteinization of serum samples. For aqueous extracts, liquid-liquid-extraction was performed after deproteination, and the aqueous layer was used. UPLC-MS data acquisition was performed in both electrospray ionization (ESI) positive and negative mode, producing four different datasets: total extract ESI-(+) (Tot^+^), total extract ESI-(–) (Tot^-^), aqueous extract ESI-(+) (Aqu^+^) and aqueous extract ESI-(–) (Aqu^-^). After preprocessing of the raw UPLC-MS data, major peaks were detected and integrated. These peak area values represented the abundance of metabolites and were used for data analysis. To detect differentially-produced metabolites, the Kruskal-Wallis test was performed, and the *p*-values were corrected by controlling the false discovery rate (FDR). As a stringent criterion, a significance level of 0.001 was used. To tentatively assign structures to these metabolites, the metabolite masses were searched using MassTRIX database search software as described in the Materials and Methods. The identities (IDs) of selected metabolites were confirmed by running authentic standards and by comparing their masses, chromatograms, MS spectra, and retention times.

### Summary statistics of the metabolites discovered and the correlation of their abundance patterns with morphometrics

A total of 4528 metabolite features were detected in at least one of the four datasets, although some metabolites were observed in more than one dataset (Table 1). Despite the stringent significance level (α = 0.001 after *p-*value adjustment), 3329 metabolite features (74%) showed significant abundance changes during metamorphosis (Table 1), highlighting the dynamic remodelling of metabolic pathways during bullfrog metamorphosis. A larger number of metabolites were detected in total extracts than in aqueous extracts because of the existence of a large number of lipophilic molecules, which is consistent with the human serum metabolome profile [13]. Of the metabolite features with significant abundance changes, 655 of them were assigned putative IDs and 89 of them were confirmed by running authentic compounds (Table 2).

**Table 1:** **The types of data generated in the experiment and summary statistics of the data analysis**^**a**^

**Serum extract**	**Stationary phase**	**ESI mode**	**Data abbreviation**	**# Metabolite features detected**	**# Significant metabolites**	**# Significant metabolites with putative IDs**	**# Confirmed metabolites**
Total	RP	ESI+	Tot^+^	2129	1648 (77%)	336	16
Total	RP	ESI–	Tot^-^	1286	1072 (83%)	212	13
Aqueous	HILIC	ESI+	Aqu^+^	693	291 (42%)	43	27
Aqueous	HILIC	ESI–	Aqu^-^	420	318 (76%)	64	33
			**Total**	**4528**	**3329 (74%)**	**655**	**89**

^a^Total extracts were prepared by complete deproteinization of serum samples and were chromatographed on a reversed-phase (RP) column to select for nonpolar metabolites. Aqueous extracts were prepared by liquid-liquid-extraction of deproteinized serum extracts and were chromatographed on a hydrophilic interaction liquid chromatography (HILIC) column to select for polar metabolites. After statistical analysis, metabolites with significant abundance changes were searched against MassTRIX database to obtain putative IDs. Subsequently, the IDs of selected metabolites were validated by running standards.

**Table 2:** Reagents used for the validation of selected metabolites

**Metabolite**	**Chemical**	**Company**	**Product number**	**MW**
1-Methyl-Histidine	1-Methyl-L-Histidine	Sigma-Aldrich	67520-50MG	169.18
Arachidonic acid	Arachidonic acid sodium salt	Sigma-Aldrich	A8798-5MG	326.45
Arginine	L-Arginine	Sigma-Aldrich	A5006-100G	174.2
C18 Sphinganine	D-*erythro*-sphinganine	Avanti Polar Lipids	60498P	301.51
C18 Sphinganine 1-Phospahte	D-*erythro*-sphinganine-1-phosphate	Avanti Polar Lipids	860536P	381.488
CDP	Cytidine 5′-Diphosphate	MP Biomedicals	0215075810	469.124
C24:1 Dihydroceramide	N-nervonoyl-D-*erythro*-sphinganine	Avanti Polar Lipids	860629P	650.113
Cer(d18:1/17:0)	N-heptadecanoyl-D-*erythro*-sphingosine	Avanti Polar Lipids	860517P	551.927
Ceramide (d18:1/16:0)	N-palmitoyl-D-*erythro*-sphingosine	Avanti Polar Lipids	860516P	537.901
C24:1 Ceramide	N-nervonoyl-D-*erythro*-sphingosine	Avanti Polar Lipids	860525P	648.097
CerP(d18:1/8:0); C8 Ceramide-1-Phosphate	N-octanoyl-ceramide-1-phosphate (ammonium salt)	Avanti Polar Lipids	860532P	522.698
cis-Aconitate	*trans*-Aconitic Acid	TCI America	A0127	174.11
Citrulline	L-Citrulline	Sigma-Aldrich	C7629-1G	175.19
CMP	Cytidine 5′-Monophosphate Disodium Salt	Sigma-Aldrich	C1006-500MG	367.16
Creatine	Creatine	MP Biomedicals	0210142225	149.1
Cystathionine	L-Cystathionine	Sigma-Aldrich	C7505-10MG	222.26
Cysteine	L-Cysteine	Sigma-Aldrich	W326305-100G	121.16
Deoxyinosine	2′-Deoxyinosine	MP Biomedicals	02101490.1	252.2
dGMP	2′-Deoxyguanosine-5′-Monophosphate Disodium Salt Hydrate	MP Biomedicals	02100561.2	391.2
Dopamine	3-Hydroxytyramine Hydrochloride	TCI America	A0305	189.64
Ethanolamine phosphate	*O*-Phosphorylethanolamine	Sigma-Aldrich	P0503-1G	141.06
Glutamine	L-Glutamine	Sigma-Aldrich	G3202-100G	146.14
Guanine	Guanine	Sigma-Aldrich	G11950-10G	151.13
Guanosine	Nucleosides Test Mix	Sigma-Aldrich	47310-U	283.24
Palmitate	Palmitic acid	Sigma-Aldrich	P0500-10G	256.42
Histidine	L-Histidine	Sigma-Aldrich	H8000-25G	155.15
Homocitrulline	L-Homocitrulline	Santa Cruz Biotechnology	sc-269298	189.21
Homocysteine	DL-Homocysteine	Sigma-Aldrich	H4628-10MG	135.18
Homoserine	L-Homoserine	TCI America	H1030	119.12
Hydroxyproline	*trans*-4-Hydroxy-L-proline	Sigma-Aldrich	H54409-100G	131.13
Hypoxanthine	Hypoxanthine	Sigma-Aldrich	H9377-25G	136.11
Inosine	Nucleosides Test Mix	Sigma-Aldrich	47310-U	268.23
Carnitine	L-Carnitine hydrochloride	Sigma-Aldrich	C0283-5G	197.66
L-DOPA	L-β-3,4-Dihydroxyphenyl-Alanine	MP Biomedicals	02101578.2	197.19
Linoleic acid	Linoleic acid sodium salt	Sigma-Aldrich	L8134-100MG	302.43
Lysine	L-Lysine	Sigma-Aldrich	L5501-25G	146.19
Methionine	L-Methionine	Sigma-Aldrich	M9625-25G	149.21
N-Acetyl-L-Aspartate	N-Acetyl-L-Aspartic Acid	Sigma-Aldrich	00920-5G	175.14
N-Arachidonoyldopamine	N-Arachidonoyl Dopamine	Cayman Chemical	90057	439.6
Nicotinamide	Nicotinamide	Sigma-Aldrich	72340-100G	122.12
Norvaline	DL-Norvaline	Sigma-Aldrich	N7502-100G	117.15
Acetylcarnitine	*O*-Acetyl-L-carnitine hydrochloride	Sigma-Aldrich	A6706-5G	239.7
Octadecenoic acid	Oleic acid	Sigma-Aldrich	O1008-1G	282.46
Oleoylcarnitine	Oleoyl-L-carnitine hydrochloride	Sigma-Aldrich	597562	462.11
Ornithine	L-Ornithine Dihydrochloride	TCI America	O0089	205.08
Pantothenate	D-Pantothenic acid hemicalcium salt	Sigma-Aldrich	P2250-5G	238.27
Proline	L-Proline	Sigma-Aldrich	P0380-100G	115.13
Riboflavin	(-)-Riboflavin	Sigma-Aldrich	R7649-25G	376.36
S-Adenosylhomocysteine	S-(5′-Adenosyl)-L-homocysteine	Sigma-Aldrich	A9384-10MG	384.41
Sphingosine 1-phosphate	Sphingosine 1-Phosphate	Sigma-Aldrich	S9666-1MG	379.47
Taurine	Taurine	Sigma-Aldrich	T0625-10G	125.15
trans-Cinnamate	*trans*-Cinnamic acid	Sigma-Aldrich	W228818-1KG-K	148.16
Trimethylglycine	Betaine aldehyde chloride	Sigma-Aldrich	B3650-2MG	137.61
Trimethyllysine	Nϵ,Nϵ,Nϵ-Trimethyllysine hydrochloride	Sigma-Aldrich	T1660-25MG	224.73
Tyrosine	L-Tyrosine	Sigma-Aldrich	T3754-50G	181.19
Uridine	Nucleosides Test Mix	Sigma-Aldrich	47310-U	244.2

**Figure 1:**
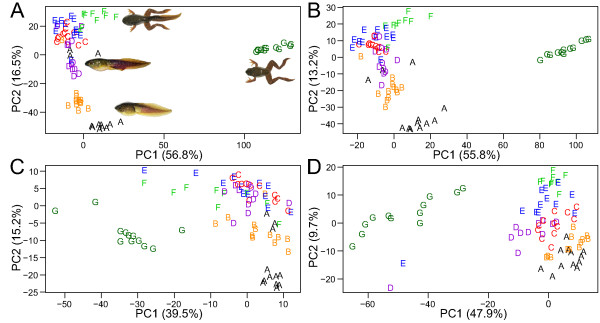
**PCA score plots of the metabolites that showed significant abundance changes.** For each dataset, PCA was performed on metabolites with statistically significant abundance changes (determined by corrected *p-*values) and the first (PC1) and second (PC2) principal components were plotted. The percentages indicate the amount of variation accounted for by each of these two components. The letters correspond to the TK stages at which the serum samples were taken from tadpoles as follows: A = TK VI–X, B = TK XII–XV, C = TK XVI–XVII, D = TK XVIII, E = TK XIX–XX, F = TK XXI–XXII, and G = > XXV. The score plots showed association between metabolite abundance change and the morphological measures (TK staging). **(A)** Tot^+^ dataset. **(B)** Tot^-^ dataset. **(C)** Aqu^+^ dataset. **(D)** Aqu^-^ dataset.

For each dataset, Principal Component Analysis was performed for those metabolites which showed significant abundance changes to determine how the metabolite abundance patterns correlate with the developmental stages of the animals from which the metabolites were extracted. The PCA plots using data from 12 individual tadpoles per group (Figure 1) showed distinct subgroups of scores, which corresponded to the different developmental stages. Developmental staging was based upon morphological criteria [12], and the data demonstrate that TK VI-X and XII-XV and froglets are readily distinguishable groups based upon metabolite features (Figure 1). TK XVI-XVII and XVIII tended to group together as late prometmorphs while a clearer progression from TK XIX-XX (start of metamorphic climax) to XXI-XXII (mid-metamorphic climax) was evident (Figure 1). This further sharpens the resolution of the distinction between postembryonic developmental stages. Since the clusters of the scores representing the froglet stage (TK > XXV) were isolated compared to other TK stage score clusters, the metabolic profile of froglets is more distinct than that of any tadpole at any previous developmental stage.

**Figure 2:**
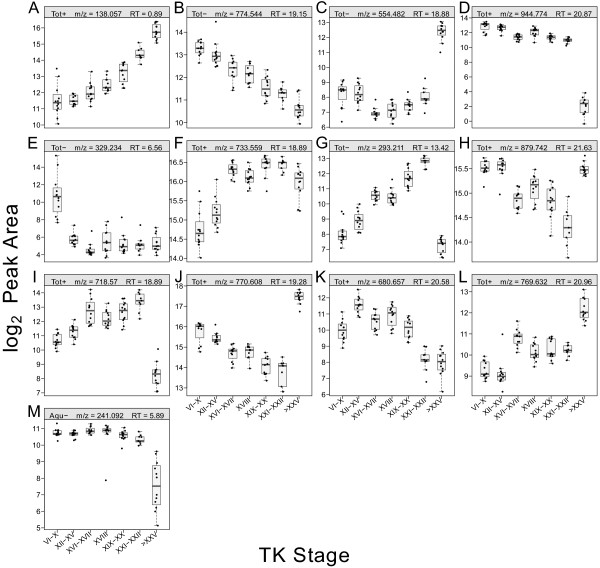
**Distinct metabolite abundance patterns that were consistently observed in the datasets.** After inspecting the abundance patterns of individual metabolites, a total of 13 different expression patterns were observed in the datasets consistently. The frequency of the observed patterns is tabulated in Table 3. **(A)** Monotonic ↑. **(B)** Monotonic ↓. **(C)** ↑ at the froglet stage (TK > XXV). **(D)** ↓ at the froglet stage (TK > XXV). **(E)** ↓ after the premetamorphic stage (TK VI–X). **(F)** ↑after the premetamorphic stage (TK VI–X). **(G)** ↑ at the metamorphic climax (TK XXI–XXII) then return to a basal level. **(H)** ↓ at the metamorphic climax (TK XXI–XXII) then return to a basal level. **(I)** ↑ at the metamorphic climax (TK XXI–XXII) followed by ↓ at the froglet stage (TK > XXV). **(J)** ↓ at the metamorphic climax (TK XXI–XXII) followed by ↑ at the froglet stage (TK > XXV). **(K)** Significant abundance change at the metamorphic climax, and the abundance remains constant at the froglet stage. **(L)** Step-wise ↑ or ↓. **(M)** Significant variation (significant unequal variance determined by the Levene’s test, *p*_adj_ < 0.01).

A box plot of log_2_ transformed peak areas *versus* TK stage ranges was created for each metabolite, and the abundance pattern produced was inspected. In total, 13 different metabolite abundance patterns were consistently observed in the datasets (Figure 2). These patterns show how tightly metabolites are regulated during metamorphosis. The frequency of these patterns was counted and tabulated (Table 3), and the top three most common classifiable patterns were: a significant decrease at the froglet stage (pattern = Figure 2D), a significant increase around the metamorphic climax and a return to basal level (pattern = Figure 2G), and a significant increase at metamorphic climax followed by a significant decrease at the froglet stage (pattern = Figure 2I). A significant decrease in the abundance of metabolites at the froglet stage accentuates how metabolically different the frog is compared to larvae upon completion of metamorphosis. A significant increase at the metamorphic climax correlates with the circulating level of THs [14]. These abundance patterns imply that the metamorphic climax is where a large fraction of metabolites exhibit an abundance change in anticipation of drastic morphological changes.

**Table 3:** Frequency of the thirteen different abundance patterns that were consistently observed in the datasets

	**Corresponding graph in Figure**2	**Number of metabolites (% of total)**			
**Pattern**		**Tot**^**+**^	**Tot**^**-**^	**Aqu**^**+**^	**Aqu**^**-**^
Monotonic ↑	A	9(0.4)	4(0.3)	3(0.4)	7(1.7)
Monotonic ↓	B	22(1.0)	33(2.6)	16(2.3)	6(1.4)
↑ at froglet	C	79(3.7)	66(5.1)	5(0.7)	7(1.7)
↓ at froglet	D	438(20.6)	286(22.2)	86(12.4)	125(29.8)
↓ after premetamorphosis	E	39(1.8)	16(1.2)	15(2.2)	3(0.7)
↑ after premetamorphosis	F	125(5.9)	17(1.3)	6(0.9)	8(1.9)
↑ at metamorphic climax then return to a basal level	G	293(13.8)	170(13.2)	30(4.3)	41(9.8)
↓ at metamorphic climax then return to a basal level	H	155(7.3)	107(8.3)	16(2.3)	2(0.5)
↑ at metamorphic climax then ↓ at froglet	I	233(10.9)	159(12.4)	7(1.0)	9(2.1)
↓ at metamorphic climax then ↑ at froglet	J	90(4.2)	44(3.4)	5(0.7)	1(0.2)
↑ or ↓ at metamorphic climax then constant	K	31(1.5)	17(1.3)	4(0.6)	5(1.2)
Step-wise ↑ or ↓	L	71(3.3)	43(3.3)	4(0.6)	3(0.7)
Unclassified		544(25.6)	324(25.2)	496(71.6)	203(48.3)
**Total**		**2129**	**1286**	**693**	**420**
Unequal variation^a^	M	94(4.4)	36(2.8)	0(0)	4(1)

^a^Unequal variation was detected by Levene’s test (*p*_adj_ < 0.01). Therefore the abundance patterns in this group include patterns from A to L.

Intriguingly, we observed some metabolites that showed a statistically significant variation in abundance patterns. For example, the abundance of the metabolite shown in Figure 2M dropped significantly at the froglet stage and also showed a large variation (heteroscedasticity). Changes in variation were also observed in our previous study [15], and poses interesting biological questions: what is causing such wide variation, what are the effects, and what is the significance of such a phenomenon? When scientists perform statistical tests, they commonly look for significant differences among data, but significant variation in data also may provide important insights.

### Remodelling of core metabolic pathways during metamorphosis

**Figure 3:**
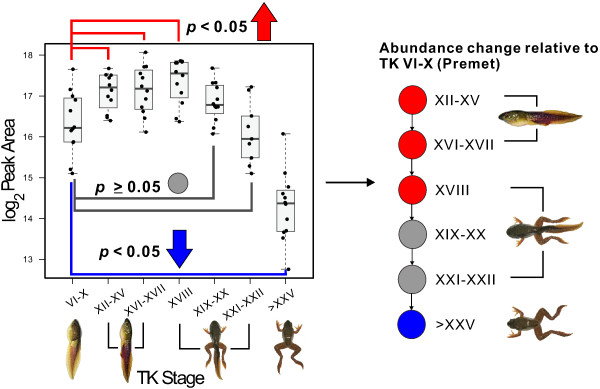
**The progression of abundance changes of metabolites.** In the metabolic pathways examined in the present study, the abundance change relative to the premetamorphic stage was illustrated using three colours: red (significant increase), grey (nonsignificant change), and blue (significant decrease).

The MassTRIX database search generated KEGG pathway maps in which the locations of query metabolites were highlighted. Using these maps, we connected and reconstructed metabolic pathway maps for the metabolites found in the present study which showed significant abundance changes. To depict the abundance changes of metabolites for each pathway relative to the premetamorphic stage at subsequent developmental stages, the direction and extent of the metabolite's abundance changes were illustrated using the colour scheme shown in Figure 3. Several components within the pathways outlined below were detected, some of which remain constant throughout this developmental period. We highlight below those metabolites and pathways for which validation with authentic standards was possible.

### Urea cycle, arginine and purine/pyrimidine metabolism

**Figure 4:**
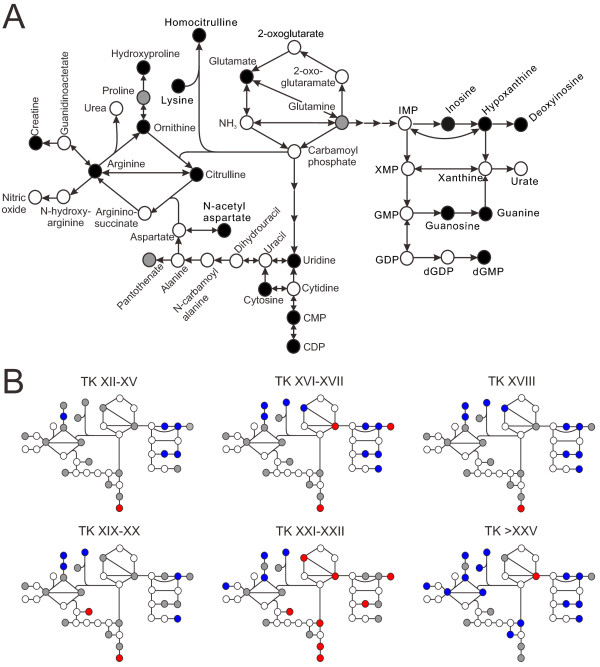
**Significant abundance changes of the metabolites in the urea cycle, arginine and purine/pyrimidine metabolism pathway.(A)** Overall abundance changes of the metabolites. Black circles indicate a significant (*p*_adj_ < 0.001) abundance change at at least one developmental stage range during metamorphosis. Grey circles indicate a nonsignificant abundance change, and white circles indicate the metabolites were not detected. **(B)** Progression of the abundance change of the metabolites relative to the premetamorphic stage (TK VI–X). The abundance levels of each metabolite at other TK stage ranges were compared to the abundance level at the premetamorphic stage (α_adj_ = 0.05). The metabolites are depicted as follows: significant ↑ (red); significant ↓ (blue); nonsignificant change (grey); and not observed (white).

The metabolic pathways for the urea cycle, arginine and purine/pyrimidine metabolism are linked to each other and many of the metabolites showed significant abundance changes during metamorphosis (Figure 4) with a general pattern of increase in abundance around the metamorphic climax, followed by a decrease at the froglet stage relative to the premetamorphic TK VI-X group (Figure 3B).

The remodelling of the nucleoside and nucleotide metabolism pathways reflects the essential roles of nucleosides and nucleotides in not only being components of DNA and RNA but also in energy metabolism. Nucleoside di- and triphosphates are substrates for ligases as well components of coenzymes [16]. As such, increased biosynthesis of ribonucleotides has been observed in tadpole liver [17,18]. It is therefore likely that the differential pattern of nucleotide metabolic pathways implies a requirement for RNA/DNA synthesis and energy during metamorphosis and tissue remodelling.

**Figure 5:**
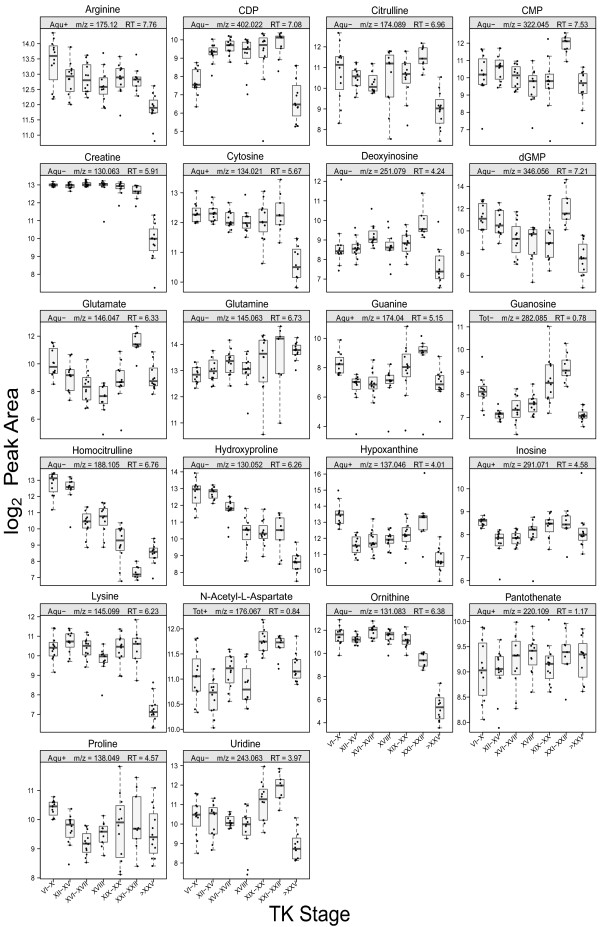
Boxplots of metabolites in urea cycle, arginine and purine/pyrimidine metabolism pathway.

**Figure 6:**
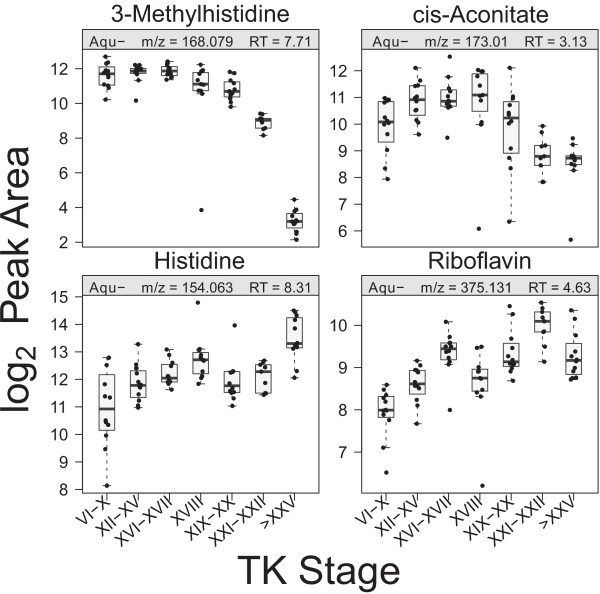
Boxplots of metabolites that were not included in any pathways.

During metamorphosis, tadpoles undergo a fasting period during which energy is provided by muscle breakdown of the tail [19,20]. Creatine acts as an energy shuttle of ATP between the mitochondrial sites of ATP production and the cytosolic sites of ATP utilization [21]. 3-methylhistidine has been shown to be a marker of muscle breakdown [22]. Both creatine and 3-methylhistidine showed a significant decrease at the froglet stage (Figures 3, 5 and 6), which correlates with the energy requirement of tadpoles during metamorphosis.

The significant changes in the abundance patterns of the metabolites in the urea cycle and arginine metabolism pathways are consistent with the extensive hepatic reprogramming and organismal reorganization from an ammonotelic larva to a ureotelic frog. At premetamorphic stages, anuran tadpoles excrete 90% of their nitrogen as ammonia [23,24], but nitrogen excretion shifts to urea rather than ammonia at metamorphic climax, and urea represents 78% of nitrogenous waste in postmetamorphic frogs [23-25]. This transition is accompanied by the activation of the urea cycle enzymes: carbamyl phosphate synthetase, ornithine transcarbamylase, argininosuccinate synthetase, argininosuccinate lyase, and arginase [26]. The differential expression of these enzymes during metamorphosis has been well-characterized [27-30]. Arginine, citrulline, and ornithine all showed a significant decrease at the froglet stage. Since these urea cycle enzymes work in a concerted manner [30] and several intermediates contribute to other metabolic pathways, it is difficult to predict the abundance patterns of urea cycle metabolites in the serum at specific developmental stages.

Homocitrulline was also observed in the data, and its abundance pattern continued to decrease until TK XXI–XXII, after which there was a slight increase at the froglet stage (Figures 3 and 5). A high level of homocitrulline in humans is associated with defects in the urea cycle, in particular with hyperammonemia, hyperornithinemia, homocitrullinuria (HHH) syndrome which is caused the deficiency of ornithine translocase, a transporter of ornithine into the mitochondria [31]. Without ornithine in the mitochondria, carbamoyl phosphate condenses with lysine to form homocitrulline. HHH syndrome is characterized by elevated plasma ornithine and ammonia levels [32]. This human disease resembles the abundance profile of ornithine found in the present study, namely an elevated level of ornithine during ammonotelic larval stages, and it is possible that the production of homocitrulline in tadpoles is due to the lack of a functional urea cycle, resulting in conditions similar to HHH syndrome.

Arginine is one of the most versatile amino acids, serving as a precursor for the synthesis of protein, nitric oxide (NO), creatine, citrulline, ornithine, and urea [33]. Of particular note is arginine’s role as a substrate in NO synthesis. NO is a radical produced from arginine by NO synthase, and this synthesis occurs in virtually all mammalian cells and tissues [34]. NO has been increasingly recognized as an important neurotransmitter and neuromodulator and has been implicated in various physiological roles in the central nervous system including nociception and olfaction [35,36], fatty acid oxidation and glucose uptake [34], as well as the release of other neurotransmitters such as norepinephrine and dopamine [37]. In *R. catesbeiana*, NO modulates the respiratory motor activity and enhances the lung burst activity [38,39]. In neurons, NO is synthesized by glutamate activation of N-methyl-D-aspartate (NMDA) receptors [40,41]. In addition to the activation of NMDA receptors to produce NO, glutamate is the major excitatory neurotransmitter with known functions in opening ion channels and stimulating inositol phospholipid cycle [42,43] and the formation of cGMP [44,45]. Glutamate was observed in our data (Figures 3 and 5), and it exhibited a significant differential abundance pattern with maximal levels at the metamorphic climax followed by a sharp decrease, a pattern similar to that found by Wiggert and Cohen [46], suggesting a higher demand for glutamate at the metamorphic climax.

### Cysteine/methionine metabolism pathway

**Figure 7:**
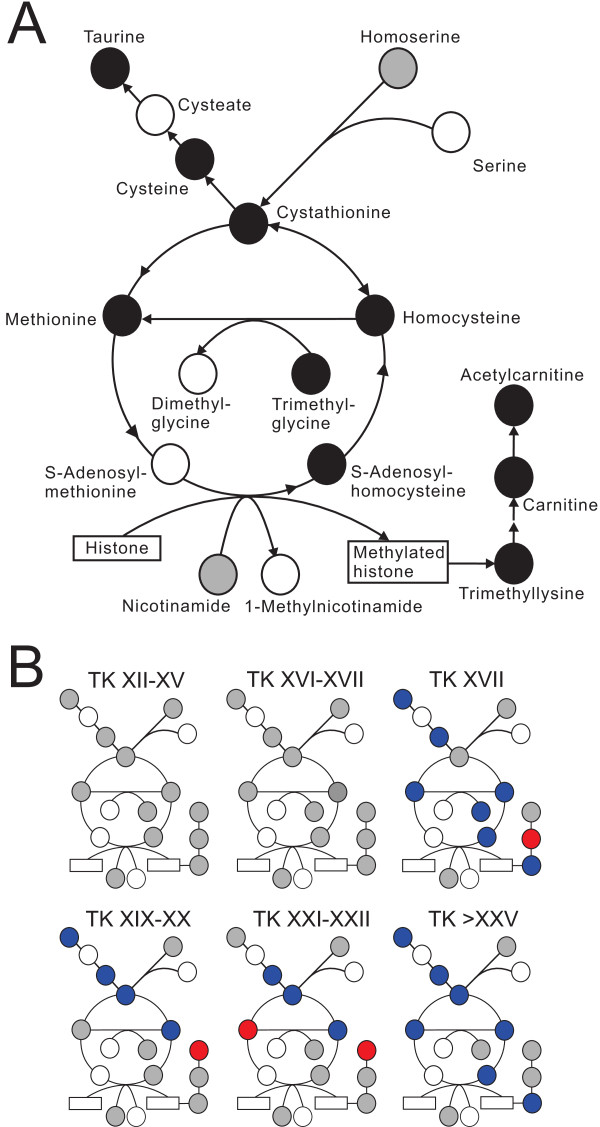
**Significant abundance changes of the metabolites in the cysteine/methionine metabolism pathway.(A)** Overall abundance changes of the metabolites. **(B)** Progression of the abundance change of the metabolites relative to the premetamorphic stage (TK VI–X). Refer to Figure 4 legend for details.

**Figure 8:**
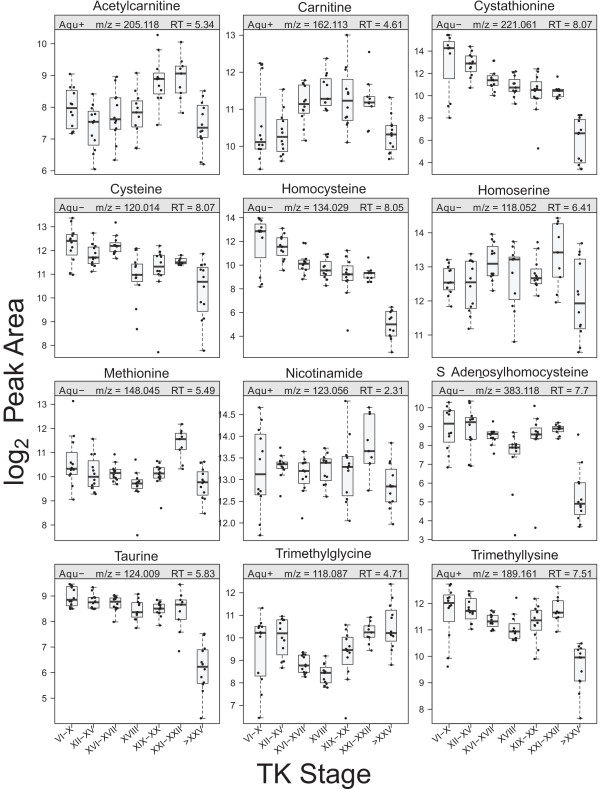
Boxplots of metabolites in the cysteine/methionine metabolism pathway.

Metabolites in the cysteine/methionine metabolism pathway showed a general decreasing pattern until the froglet stage (Figures 7 and 8). Both cysteine and methionine are important antioxidant in biological systems. Cysteine is a substrate for the formation of glutathione, and methionine acts as an endogenous antioxidant in proteins [47]. Another important aspect of this metabolic pathway is the production of S-adenosylmethionine (SAM), the principal biological methyl donor. Upon methyl group transfer, SAM is converted to S-adenosylhomocysteine (SAH), and the SAM/SAH ratio is considered to be an indicator of cellular methylation capacity [48]. Methylation plays critical roles in epigenetics, reprogramming, and cancer, and histone methylation has been shown to regulate the action of TH receptor (TR) in *Xenopus tropicalis* upon T_3_ treatment [49,50]. SAM was not detected in the present study, but SAH was detected, and its abundance dropped dramatically at the froglet stage. SAH inhibits the action of most SAM-dependent methyltransferases, and it has been suggested that metabolite modulation of DNA methyltransferases occurs mainly through SAH in many cell types [51].

Trimethyllysine found in the present study suggests the importance of histone methylation during metamorphic reprogramming. Among the possible histone modifications, methylation represents a complex type of modification that targets primarily histone H3, in which arginine and lysine residues can be mono-, di-, or trimethylated [52]. The importance of histone modification during metamorphosis has been demonstrated – Matsuura *et al.*[50] showed that TRs induce histone modifications to activate transcription during larval intestinal cell death, and adult stem cell development in *X. tropicalis*, and Bilesimo *et al.*[49] observed gene and tissue-specific patterns of histone methylation upon TH treatment of premetamorphic *X. tropicalis* tadpoles in the tail fin and the brain. TH treatment decreased the level of a repressive marker, Me3H3K27, and increased the level of an activation marker, Me3H3K79, thereby initiating transcription of TH target genes in *X. tropicalis* intestine [50] and tail fin [49]. Interestingly, both SAH and trimethyllysine showed similar abundance patterns - a decrease until TK XVII, followed by an increase until TK XXI–XXII, and then a sharp decline at the froglet stage (Figures 7 and 8).

Trimethyllysine is also a precursor of carnitine and acetylcarnitine. Carnitine acts as a shuttle to transport long-chain fatty acids from the cytosol into the mitochondria during lipid catabolism for the generation of metabolic energy [53], and both carnitine and acetylcarnitine showed a general increase at the metamorphic climax (Figures 7 and 8), suggesting increased lipid mobilization at this time in development.

Taurine, a precursor of taurocholate, exhibited constant levels throughout development with a significant decrease at the froglet stage (Figures 7 and 8). Taurine has many roles in metabolism such as osmoregulation, modulation of Ca^2+^ dependent processes, and antioxidation [54]; however, the significance of the regulation of this metabolite is not clear.

### Lipid metabolism

**Figure 9:**
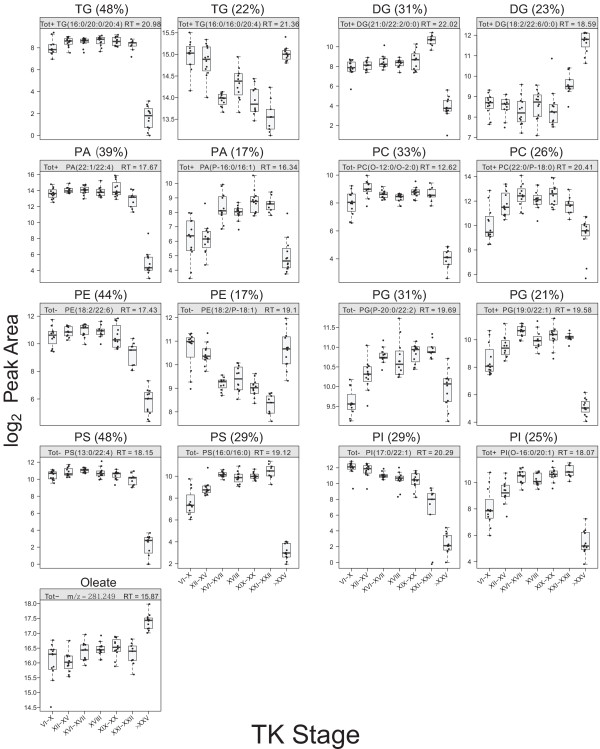
**The two most common abundance patterns for the lipid classes observed.** Each lipid class exhibited common abundance patterns, and the two most common abundance patterns are presented. In each graph, the percentage values correspond to the fraction of lipids that exhibited the abundance pattern of the graph. In most lipid classes, the most common abundance pattern was a drop at the froglet stage. “O-“, alkyl ether linkage; “P-“, (1Z)-alkenyl ether (neutral plasmalogen) species.

Most lipid molecules were detected in the total metabolite extracts, and phospholipids were predominant, which is consistent with the human metabolome profile [55]. However, a large number of structural isomers are possible for each lipid metabolite, so we were only able to identify lipid classes (Table 4 and Figure 9). Each lipid class showed specific abundance patterns, but the most common abundance pattern for these lipid metabolites was a sharp drop at the froglet stage (Table 4 and Figure 9).

**Table 4:** Summary of the lipid metabolites discovered

**Lipid**	**Abbreviation**	**Number identified**	**Most common pattern**
Triglyceride	TG	79	↓ at froglet
Diglyceride	DG	13	↑ at climax then ↓ at froglet
Phosphatidic acid	PA	36	↓ at froglet
Phosphatidylcholine	PC	27	↓ at froglet
Phosphatidylethanolamine	PE	18	↓ at froglet
Phosphatidylglycine	PG	29	↑ at climax
Phosphatidylserine	PS	62	↓ at froglet
Phosphatidylinositol	PI	28	↓ at froglet

Most of these lipid metabolites were found in Tot^+^ or Tot^-^. For many lipid metabolites, structural isomers were possible, therefore only the type of the lipid molecule (*e.g.* TG) was used as the ID.

Little is known about lipid metabolism during frog metamorphosis. Triglycerides (TG) constitute the majority of the fat body in anurans [56], and TG was the most common among the lipid metabolites identified in the present study (Table 4). Interestingly, many of these lipids showed two common abundance patterns: a decrease after the metamorphic climax or an increase until the metamorphic climax followed by a decrease (Figure 9). A study by Sawant and Varute [57] showed a similar lipid profile in *R. tigrina*, in which the total lipid and TG concentrations also increased until the metamorphic climax followed by a sharp decrease. This trend may be due to increased mobilization of lipids during metamorphosis to provide the energy required for the remodelling of organs and tissues as the animals progress to a state at the metamorphic climax, where they cease to eat until metamorphosis is completed. The known effects of THs on lipid metabolism include enhanced catabolism and an increase in the synthesis and mobilization of TGs stored in adipose tissue [58,59], and the detection of carnitine and acetylcarnitine, as shown in the cysteine/methionine metabolism pathway, corroborates this idea.

Another interesting observation was the discovery of 62 phosphatidylserine (PS) forms (structural isomers could not be differentiated) of which 48% showed a significant decrease after the metamorphic climax (Table 4 and Figure 9). PS accounts for 5-20% of the total phospholipids in the cell membrane and is located on the inner leaflet of the lipid bilayer [60]. PS on the surface of red blood cells is a biomarker for apoptosis [61] as the appearance of PS on the cell surface serves as a mechanism for macrophages to recognize apoptotic cells due to changes in surface hydrophobicity. As macrophages increase in number at the metamorphic climax, it is likely that an abundance change of PS may correlate with the extent of apoptosis occurring during metamorphosis in *R. catesbeiana*.

**Figure 10:**
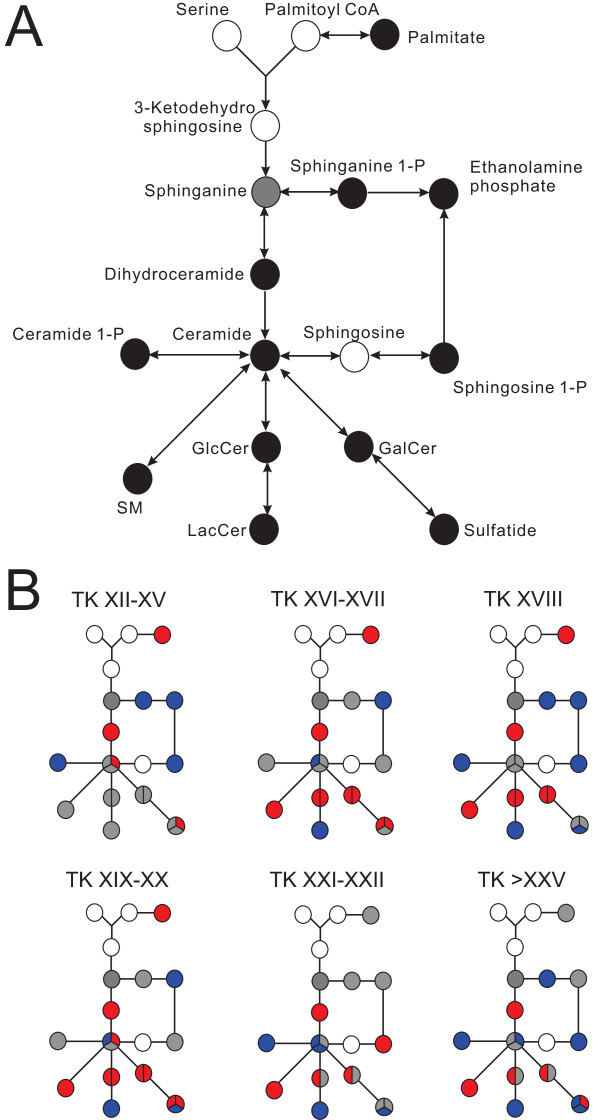
**Significant abundance changes of the metabolites in the sphingolipid metabolism pathway.** Glucosylceramide (GlcCer) and galactosylceramide (GalCer) are structural isomers and hence cannot be differentiated, but both metabolites are converted to distinct metabolites, so they are depicted separately. The divided circles indicate variants of that metabolite (different chain length) were detected. Abbreviations: GalCer: galactosylceramide, GlcCer: glucosylceramide, LacCer: lactosylceramide, SM: sphingomyelin. **(A)** Overall abundance changes of the metabolites. **(B)** Progression of the abundance change of the metabolites relative to the premetamorphic stage (TK VI–X). Refer to Figure 4 legend for details.

**Figure 11:**
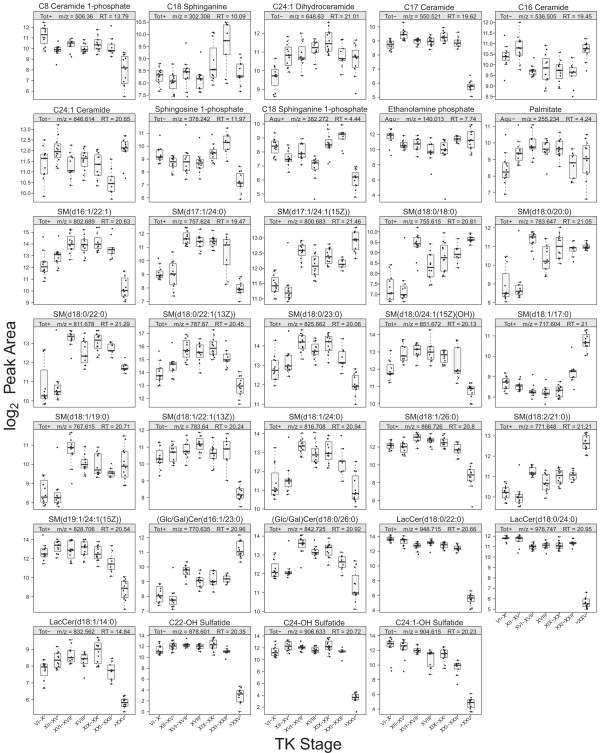
**Boxplots of metabolites in the sphingolipid metabolism pathway.** “d”, 1,3-dihydroxy long-chain base.

Despite the possibility of a large number of structural isomers, we were able to identify the key metabolites in the sphingolipid metabolism pathway (Figures 10 and 11) by comparing them to authentic compounds because the database search yielded either one or a few hits for these metabolites. What is unique about the sphingolipid metabolism pathway is that the metabolites in this ubiquitous evolutionarily conserved pathway are implicated in various signal transduction pathways and, unlike the classical cAMP signalling cascade, the sphingolipid metabolism pathway is more complex because enzymes are intimately related to each other, the metabolites are recycled in the pathway, and interconversions are common [62].

The two key metabolites of the pathway are ceramide and sphingosine 1-phosphate (S1P). These two metabolites have been known to exert opposing effects in biological systems - ceramide promotes senescence, differentiation, apoptosis and cell-cycle arrest whereas S1P induces proliferation, mitogenesis, inflammation, migration, angiogenesis, and protection from apoptosis [63]. We were able to identify three ceramides with different chain lengths: C16, C17 and C24:1 (Figure 10). The pathway begins with the condensation of serine and palmitoyl-CoA, generated from palmitate, a C-16 fatty acid, and C16 ceramide is the most predominant form of ceramides and has been shown to induce activation-induced cell death in Ramos B-cells [64].

S1P and the kinases that produce it have emerged as crucial regulators of numerous biological processes [13] and their actions are evolutionarily conserved [62]. S1P is produced by sphingosine kinase and is a ligand for five G-protein-coupled receptors leading to activation or inhibition of downstream enzymes in numerous signalling pathways including extracellular signal-related kinase (ERK), Jun amino terminal kinase (JNK), the small GTPases of the Rho family (Rho and Rac), phospholipase C (PLC), adenyl cyclase-cyclic AMP, and phosphatidylinositol 3-kinase (PI3K) [62]. S1P also promotes cell migration, angiogenesis, calcium homeostasis, and DNA synthesis, and it is highly likely that this metabolite plays crucial roles during remodelling in metamorphosis [62,65]. Though not as well-studied as S1P, ceramide 1-phosphate (C1P) has also been reported to promote mitogenesis and block apoptosis [66].

The progressive changes in the abundance of metabolites in the sphingolipid metabolism pathway did not show a clear pattern, and this might be because of the recycling and interconversion of the metabolites in this pathway. C17 ceramide levels decreased significantly at the froglet stage whereas C16 and C24:1 ceramides showed a significant increase at the froglet stage (Figures 10 and 11). S1P level showed an increase around the metamorphic climax, peaking at TK XXI–XXII, followed by a sharp decrease at the froglet stage. This pattern, resembling that of circulating TH levels, also supports the possible role of S1P as an important regulator of metamorphosis, as most drastic remodelling occurs at the metamorphic climax.

### Eicosanoid metabolism pathway

**Figure 12:**
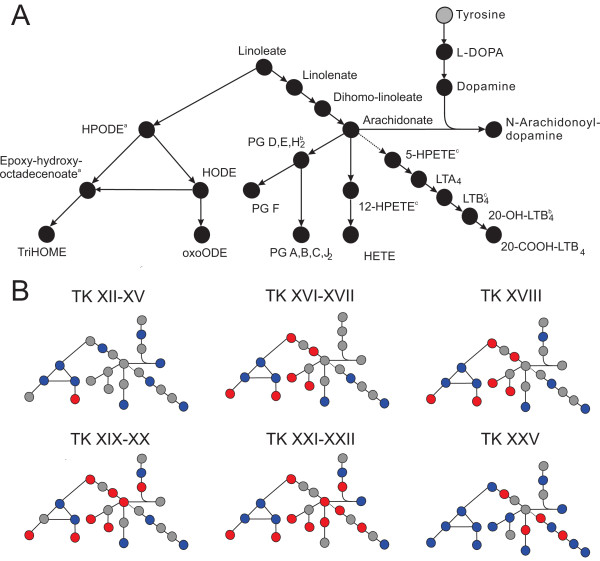
**Significant abundance changes of the metabolites in the eicosanoid metabolism pathway.** The superscripts indicate that the metabolites have the same mass and cannot be differentiated. **(A)** Overall abundance changes of the metabolites. **(B)** Progression of the abundance change of the metabolites relative to the premetamorphic stage (TK VI–X). Refer to Figure 4 legend for details. Metabolites with the same superscript letter (a, b, or c) share the same mass and cannot be distinguished from each other.

**Figure 13:**
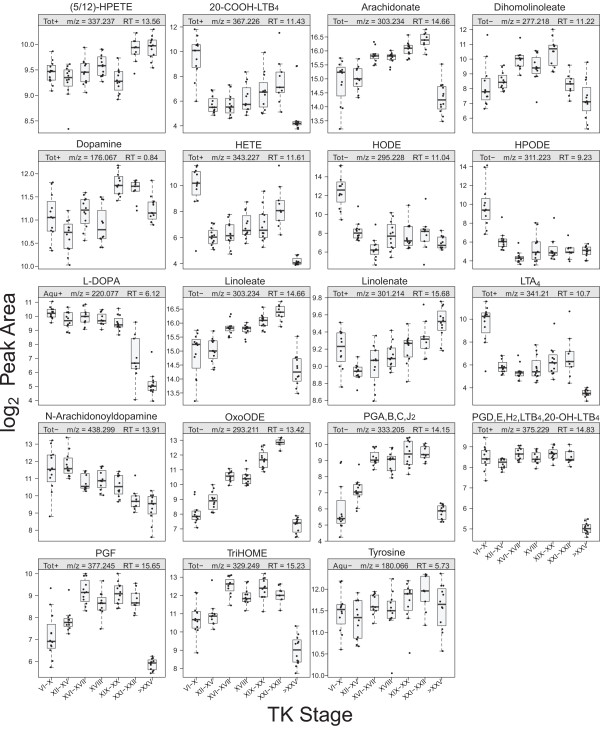
Boxplots of metabolites in the eicosanoid metabolism pathway.

Arachidonic acid-derived eicosanoids, including prostaglandins (PG) and leukotrienes (LT), act as signalling molecules that control diverse biological responses such as vascular homeostasis and inflammatory responses to tissue remodelling [67]. The metabolites in the eicosanoid metabolism pathway showed a significant abundance change (Figures 12 and 13). PG A, B, C, and J_2_ could not be distinguished because they are structural isomers. Similarly, other groups of metabolites were indistinguishable including PG D, E, H_2_, LTB_4_, and 20-OH-LTB_4_. Anurans have substantially different immune systems at the larval and frog stages [68]. It has been hypothesized that the development of molecules specific to the frog stage (adult hemoglobin, adult-type keratin, the urea cycle enzyme L-arginase, etc.) could elicit self-destructive immune responses during metamorphosis [69]. To avoid this, amphibians self-destruct their lymphocytes [68], which is supported by the fact that amphibian metamorphosis is not characterized by autoimmune tissue destruction. In *Xenopus laevis*, a decline in lymphocytes during metamorphosis has been observed in the spleen, thymus, and liver [69-71]. This hypothesized remodelling of the immune system in anurans may explain the dynamic change in the eicosanoid metabolism pathway that occurs during metamorphosis.

Eicosanoids play an integral role in immunity, differentiation, cell proliferation, migration, and antigen presentation [67], and arachidonic acid is the central molecule that gives rise to other eicosanoids [72]. Arachidonic acid induces apoptosis [73,74] and the maximal level at the metamorphic climax suggests a possible role of arachidonic acid in tissue remodelling during metamorphosis. Hydroxyeicosatetraenoic acid (HETE) and hydroperoxyeicosatetraenoic acid (HPETE), also products of arachidonic acid formed during inflammation, regulate angiogenesis [75]. HETE promotes angiogenesis whereas HPETE inhibits angiogenesis. Arachidonic acid levels increased significantly, peaking at the metamorphic climax and decreasing significantly at the froglet stage (Figures 12 and 13). HETE showed a significant decrease after the premetamorphic stage, increasing gradually until the metamorphic climax, then dropping significantly at the froglet stage (Figures 12 and 13). HPETE remained constant and increased at the metamorphic climax, and the increased level remained at the froglet stage (Figures 12 and 13). The role of HETE and HPETE in angiogenesis also implies a role in tissue remodelling during metamorphosis. The differential abundance patterns of these two metabolites (Figures 12 and 13) suggest they may work in a concerted manner for vascularization throughout metamorphosis.

In addition to the eicosanoid metabolism pathway, we detected tyrosine, L-3,4-dihydroxyphenylalanine (L-DOPA), dopamine, and N-arachidonoyldopamine (NADA) (Figures 12 and 13). Tyrosine did not show a significant abundance change. The abundance of L-DOPA dropped significantly at TK XIX–XX until the froglet stage. L-DOPA is a precursor for catecholamines including dopamine, norepinephrine, and epinephrine that are implicated in various physiological processes and the hormonal control of metamorphosis, and the dropping level of L-DOPA around metamorphic climax may indicate the requirement of L-DOPA to synthesize catecholamines to execute metamorphosis. Dopamine showed a pattern of a general increase around the metamorphic climax followed by a decrease at the froglet stage (Figures 12 and 13). This pattern may be explained by the role of dopamine as an inhibitor of the release of prolactin (PRL), an antimetamorphic hormone [76]. It has been suggested that the role of PRL is to counteract high concentrations of THs at the metamorphic climax to coordinate the subsequent transformations of organs and tissues. The inhibitory effect of dopamine on PRL release may be another way of controlling the circulating level of THs in order to tightly regulate the completion of metamorphosis. The levels of tyrosine did not change significantly during metamorphosis. Tyrosine is a precursor for the synthesis of THs in the thyroid gland, but we did not detect T_3_ or T_4_ in the present study. This is likely because most of the circulating THs in plasma are bound to TH binding proteins [2], and after complete deproteinization of serum samples, THs were removed along with TH binding proteins.

NADA is an endogenous lipid of the central nervous system and acts on both transient receptor potential vanilloid type 1 (TRPV1) and cannabinoid type 1 (CB1) receptor. The novel properties of NADA as an antioxidant and neuroprotectant have been discovered [77], and NADA has been shown to induce TRPV1-dependent cell death in neurone-like cells independent of caspase activity [78]. Studies indicate that the CB1 receptor is implicated in brain and neuronal development [79]. Taken together, this may indicate that NADA may affect brain and neuronal development during metamorphosis.

### Other metabolites

Outside the aforementioned metabolic pathways, we detected cis-aconitate, histidine, and riboflavin (Figure 6). The abundance pattern of *cis*-aconitate increased around TK XVIII followed by a gradual decrease until the froglet stage, possibly representing the metabolic status of the citric acid cycle as *cis-*aconitate is an intermediate in the conversion of citrate to isocitrate. Riboflavin exhibited a peculiar abundance change, increasing until TK XVI–XVII followed by a decrease at stage XVIII then increasing again until TK XXI–XXII, finally plummeting at the froglet stage (Figure 6). Riboflavin is a versatile metabolite and is the core component of flavoproteins. Flavoproteins have various roles in redox reactions, signal transduction, programmed cell death, regulation of biological clocks, and light-dependent repair of DNA damage [80]. The requirement for the versatile actions of flavoproteins likely increases during metamorphic remodelling.

## Conclusions

Using a validated metabolomics approach, we were able to identify key metabolites and metabolic pathways - arginine and purine/pyrimidine, cysteine/methionine, sphingolipid, and eicosanoid metabolism and the urea cycle - that are significantly remodelled during bullfrog metamorphosis. Of particular note is the prominent role of lipids providing a new mechanistic avenue in the control of this important postembryonic developmental process. Since metamorphosis is hormonally-controlled, the discoveries herein draw attention to systems that present as strong candidates for TH-mediated coordination of organism remodelling.

## Methods

### Animals and serum collection

*R. catesbeiana* tadpoles used in the present study were caught locally and were maintained in accordance with the guidelines of the Canadian Council on Animal Care and the University of Victoria (Permit # 2010-030). Euthanasia was performed using buffered tricaine methanesulfonate (MS-222) (Syndel Laboratories Ltd., Vancouver, Canada) at either 0.1% (w/v) for tadpoles or 1% (w/v) for froglets. The solutions contained 25 mM of sodium bicarbonate and were freshly prepared in dechlorinated tap water immediately before use. Animals were individually staged according to TK staging [12]. To obtain blood, a deep, vertical incision was made on the tail musculature close to the abdomen using a sharp razor blade. Blood was collected using a pipettor and transferred to a microcentrifuge tube. The blood was allowed to coagulate for 15 min at room temperature and then centrifuged at 4°C at 16,000 × *g* for 10 min. The serum was separated from the cell pellet, flash frozen in liquid nitrogen and stored at -80°C until further processing.

Seven different TK stage ranges were used in the present study: VI–X, XII–XV, XVI–XVII, XVIII, XIX–XX, XXI–XXII, and > XXV. For each TK stage range, 12 biological replicates were obtained, hence there were 84 samples in total. Because the volumes of three of the serum samples obtained from metamorphs at TK XXI–XXII were insufficient, these samples were not tested, and the number of biological replicates for stage TK XXI–XXII was 9. Therefore, a total of 81 serum samples were analyzed in the present study.

### “Total” metabolite extraction

To reduce the possibility of systematic error, the samples were processed in a randomized order. Twenty-five μL of serum from each tadpole were mixed with 25 μL of water in a 0.65 mL-microcentrifuge tube, and 500 μL of acetonitrile was added. The tube was vortexed vigorously and then placed on ice for 30 min to completely precipitate proteins. Following centrifugation at 4°C at 12,000 × *g* for 10 min, 500 μL of the supernatant were transferred to a V-tapered sample vial and then dried in a Savant SPD1010 SpeedVac concentrator (Thermo Electron, Milford, MA, USA). The residues were reconstituted in 40 μL of 20% isopropanol, of which 7.5 μL were injected for each UPLC-mass spectrometry (UPLC-MS) run.

### Liquid-liquid extraction of polar (aqueous) metabolites

Fifty μL of each tadpole serum sample were mixed with 500 μL of methanol in a 1.5-mL Eppendorf tube. After 15 s × 2 vortex-mixing, the tube was placed on ice for 30 min and centrifuged as above. Following centrifugation, 500 μL of the supernatant were transferred to a 1.5-mL Eppendorf tube and mixed with 175 μL of water and 350 μL of chloroform. Following a brief vortexing, the tube was centrifuged at 4°C at 12,000 × g for 10 min to separate the whole phase into aqueous (upper) and organic (lower) phases. Five hundred μL of the aqueous phase were carefully transferred to a V-tapered sample vial and dried in the same SpeedVac concentrator. The residue was reconstituted in 50 μL of 90% acetonitrile and 5 μL were injected for UPLC-MS.

### UPLC-MS

All data files were acquired on an Acquity UPLC system coupled to a Synapt Q-TOF mass spectrometer (Waters, Milford, MA, USA). UPLC-MS was performed using two columns: a Waters BEH C18 (2.1 mm I.D. × 100 mm, 1.7 μm) column for the total metabolite extracts and a Waters BEH Amide (2.1 mm I.D. × 100 mm, 1.7 μm) column for the separation of very polar metabolites. On the C18 column, a binary solvent gradient elution was used to chromatograph the metabolites with 0.01% formic acid in water as mobile phase solvent A and isopropanol-acetonitrile (1:1, v/v) containing 0.01% formic acid as mobile phase solvent B. Column temperature was kept at 45°C, and the flow rate was 0.25 mL/min. The binary gradient was from 8% to 40% solvent B in 5 min, 40% to 100% solvent B in 17 min, and then 100% solvent B for 3 min. The column was re-equilibrated with 8% solvent B for 5 min before the next injection. With the Waters Amide column, a binary solvent gradient elution was used to separate the metabolites with acetonitrile containing 0.01% formic acid as solvent A of the mobile phase and 0.01% formic acid in water as solvent B of the mobile phase. Column temperature was 30°C, and the flow rate was 0.25 mL/min. The binary gradient was 10% to 70% solvent B in 12 min, 70% solvent B for 2 min and then the column was reconditioned with 10% solvent B for 6 min before the next injection.

The eluted metabolites were ionized by electrospray ionization (ESI) and detected in both the positive and negative ion modes over the mass range m/z 100-1000. This resulted in 4 UPLC-MS datasets per sample (*i.e.*, 4 UPLC-MS runs per sample were carried out): total extract ESI(+)(Tot^+^), total extract ESI(–)(Tot^-^), aqueous extract ESI(+)(Aqu^+^), and aqueous extract ESI(–)(Aqu^-^). The typical ESI-MS parameters included an ESI spray voltage of 3-3.2 kV, desolvation gas (N_2_) flow of 750-800 L/h, a temperature of 350°C, drying gas (N_2_) flow of 50 L/h and temperature of 130°C, sampling cone voltage of 35 V, extraction cone voltage of 4 V, and data acquisition rate of 0.25 s. The background argon gas in the collision cell was kept at 0.8 mL/min. A lock-mass spray (50 pg/μL leucine enkephaline in 60% isopropanol at 5 μL/min) was employed to ensure the mass accuracy of the TOF throughout the UPLC-MS runs.

### Data preprocessing

Raw UPLC-MS data were converted to the netCDF files using the Waters Databridge translation utility. The resulting data files from each dataset were then processed using the XCMS package [81], an R package which performs non-linear correction of retention time (RT) shifts. Peak detection and integration was performed using the centWave algorithm [82]. RT shift correction was achieved considering at least 200 peak groups. After two iterations of peak grouping, peak filling was done using the “fillPeaks” routine of the XCMS package. Finally, a data matrix was generated from each UPLC-MS dataset and exported into Microsoft Excel. After removal of the significant background noise signals observed in each UPLC-MS blank run and manual de-isotoping, the individual data matrices were saved as two-dimensional (m/z-RT pair vs. peak area) data tables amenable to subsequent statistical analyses.

### Statistical analysis

**Figure 14:**
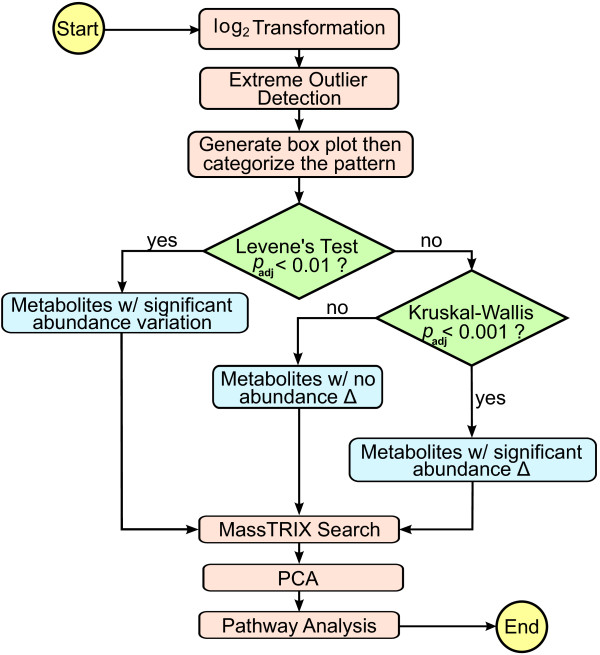
**Data analysis work flow.** After preprocessing of the data, it was analyzed with the indicated decision points.

All statistical analyses were performed using the R programming language [83]. The data analysis work flow is presented in Figure 14. The peak area values in the datasets were log_2_ transformed to reduce variance and to make the skewed distributions of the data more symmetric. One sample from the Tot^-^ set produced poor signal for most metabolites and was removed from the analysis. Box plots were made for all metabolites, and the abundance pattern of each graph was inspected thoroughly. The patterns of the graphs were classified into one of thirteen categories that consistently appeared (Table 3). To identify metabolites with differential abundance patterns at different TK stages, the Kruskal-Wallis test was performed for each metabolite under the null hypothesis of the same median log_2_ peak integration values across all the TK stages. The Kruskal-Wallis test assumes that the distributions of data for each metabolite at different TK stages have identical shapes, implying that these distributions have equivalent variances. Violation of this constant variance assumption results in inaccurate *p-*values, hence unreliable results. To prevent this, each metabolite was tested for equal variance using Levene’s test using a median as the central location parameter of a distribution. The *p-*values obtained after Levene’s test were adjusted for multiple comparisons by controlling the FDR as proposed by Benjamini and Hochberg [84]. After the adjustment, metabolites with *p*_adj_ <0.01 were determined to have significantly different variances, and these metabolites were separated prior to the Kruskal-Wallis test. After performing the Kruskal-Wallis test, the *p-*values were adjusted by controlling the FDR using the method by Benjamini and Hochberg [84], and metabolites showing highly statistically significant abundance changes (*p*_adj_ <0.001) were chosen for further database search. PCA was performed to display the relationship between TK staging and the abundance profiles of significant metabolites. For a data matrix, *n* × *p* where *n* = samples (tadpoles) and *p* = significant metabolites, PCA was performed by centering the data matrix by column-wise medians and then singular value decomposition of the median-centered data matrix. Scaling was not performed because of the wide range of metabolic abundance changes.

### Database search, identification of metabolites and pathway construction

The metabolite features whose abundance profiles showed significant heteroscedacity at a certain TK stage or significant abundance changes were searched against the MassTRIX ver. 3 webserver [85] (http://masstrix3.helmholtz-muenchen.de/masstrix3/). For the ESI^+^ generated datasets (Tot^+^ and Aqu^+^), [M + H]^+^ and [M + Na]^+^ were selected as possible adducts while [M-H]^-^ was chosen as a possibility for the ESI^-^ generated data (Tot^-^ and Aqu^-^). The allowable mass error was set to 0.02 Da, and KEGG/HMDB/LIPID MAPS [86-88] without isotopes was selected as the database. Because *Rana catesbeiana* was not available as a choice of organism, *Homo sapiens* was chosen due to the completeness of the database and the similarities in genetic diversity and metabolomic systems. In the optional pathway analysis field, 90 different KEGG pathway IDs were pasted in order to obtain a comprehensive coverage of the possible locations of the metabolites in metabolic pathways. These pathways included the citric acid cycle, fatty acid synthesis, steroid hormone biosynthesis, amino acid metabolism and degradation, etc. When there were multiple hits for the possible IDs of one metabolite, the most likely ID was inferred based on the chemical profile of the metabolite (retention time, ESI mode, existence of similar metabolites, etc.). We focused only on those masses with only one possible ID or where all but one ID had been eliminated by chemical profile evaluation. Using the KEGG pathway maps in which the locations of query metabolites were highlighted, we connected and constructed metabolic pathway maps. For each metabolite, the integration values at the indicated TK stage ranges were compared to the values at the premetamorphic stage as a control, using the nonparametric multiple comparison procedure for unbalanced one-way factorial design proposed by Gao *et al.*[89]. The *p-*values were FDR-corrected, and a significance level of 0.05 was used.

### Validation of selected metabolites

Authentic compounds for the selected metabolites were obtained as shown in Table 2. These compounds were prepared and diluted to a final concentration of 10 μg/mL in 20% isopropanol for the metabolites observed in total extracts and 90% acetonitrile for the metabolites observed in aqueous extract. The standards were run and analyzed under the same conditions as described in the UPLC-MS section.

## Abbreviations

C1P: Ceramide 1-phosphate; CB1: Cannabinoid type 1; ESI: Electrospray ionization; FDR: False discovery rate; HETE: Hydroxyeicosatetraenoic acid; HHH: Hyperammonemia, hyperornithinemia, homocitrullinuria; HPETE: Hydroperoxyeicosatetraenoic acid; ID: Identities; L-DOPA: L-3,4-dihydroxyphenylalanine; LT: Leukotriene; MS: Mass spectrometry; NADA: N-arachidonoyldopamine; NMDA: N-methyl-D-aspartate; NO: Nitric oxide; PCA: Principal components analysis; PG: Prostaglandin; PRL: Prolactin; PS: Phosphatidyl serine; Q-TOF: Quadrupole time-of-flight; R.: Rana; RT: Retention time; S1P: Sphingosine 1-phosphate; SAH: S-adenosylhomocysteine; SAM: S-adenosylmethionine; TH: Thyroid hormone; TG: Triglyceride; TK: Taylor Kollros; TR: Thyroid hormone receptor; TRPV1: Transient receptor potential vanilloid type 1; UPLC: Ultra-performance liquid chromatography.

## Competing interests

The authors declare that they have no competing interests.

## Authors’ contributions

TI isolated the serum, performed the mass spectrometry, data, and statistical analyses, and drafted the manuscript. JH performed mass spectrometry and data analysis. CB participated in the design of the study and provided reagents/materials/analysis tools. ML participated in the experimental design and statistical analysis. CH conceived of the study, and participated in its design and coordination and helped draft the manuscript. All authors read and approved the final manuscript.
